# Supplementary material for: Augmenting electronic health record data with social and environmental determinant of health measures to understand regional factors associated with asthma exacerbations
Source: PLOS Digit Health. 2025 Jun 23;4(6):e0000677. doi: 10.1371/journal.pdig.0000677 (PMC12184914; doi:10.1371/journal.pdig.0000677)
Supplement: S1 Text — (DOCX) [file pdig.0000677.s001.docx]

**S1 Text. Supplementary Methods.**

**Inclusion and Exclusion Criteria**

De-identified EHR data was obtained for 86,787 Penn Medicine patients who had any asthma encounter, according to presence of a J45* International Classification of Diseases (ICD)-10 code, between 1/1/2017 and 12/31/2020. Patients from this dataset were selected according to the following inclusion criteria: 1) at least 18 years old at first encounter during the study period, 2) at least one instance of a primary asthma diagnosis based on ICD-10 of J45* in any encounter type during the study period, 3) at least one year followed with at least two encounters during the study period, and 4) at least one prescription for a short-acting β_2_-agonist (SABA) (S1 Table) during the study period, as U.S. national asthma management guidelines at that time recommended SABA treatment for managing intermittent and persistent asthma in individuals of ages 12 years and older [1]. We excluded patients with an ICD-10 code corresponding to cystic fibrosis (E84*, N = 123), patients with greater than 10 exacerbations during the study period (N = 70), and patients with incomplete EHR data (see EHR-derived variables, N = 303).

**EHR-Derived Variables**

*Demographic variables*

Several demographic variables were obtained from codified EHR fields, cleaned, and included as independent variables in our analysis. Age was determined as the age at first encounter during the study period and categorized as: *18-34, 35-54, 55-74*, or *75+* years. Sex, race, ethnicity, and smoking status were determined using the most recent entry in the EHR at the time the dataset was extracted (May 2023). Race was categorized as: *White, Black,* or *unknown/other.* Ethnicity was captured as *Hispanic/Latino* and *non-Hispanic/Latino*. Smoking status was categorized as: *never smoked, ever smoked,* or *current smoker*. A small number of patients reported *passive exposure*, so this category was combined with *never smoked*. BMI and health insurance type were determined using the most recent encounter during the study period. If a patient had multiple encounters on the day of their most recent visit, BMI and health insurance type were defined as the most frequent non-missing entry on that day, or, in the case of ties, as the most frequent non-missing entry across all encounters during the study period. If the most recent encounter reported BMI or health insurance type as missing, their value was imputed as the most frequent non-missing entry across all encounters during the study period. All ties were broken by recency. BMI was categorized using standard CDC categories as: *not overweight or obese* (<25.0 kg/m^2^), *overweight* (25.0 to <30.0 kg/m^2^), *class 1 obesity* (30.0 to <35.0 kg/m^2^), *class 2 obesity* (35.0 to <40.0 kg/m^2^), and *class 3 obesity* (≥40.0 kg/m^2^). BMI values less than 10.0 kg/m^2^ or greater than 105.0 kg/m^2^ were excluded to reduce bias from potential data entry errors. Health insurance type was categorized as: *private, Medicaid,* or *Medicare*. Years followed was computed as the number of days between the first and last encounter for each patient divided by 365.25. Patients with missing data for any of the above variables (i.e., age, sex, ethnicity, health insurance type, BMI, smoking status) were excluded; those with missing race were included and assigned to the *unknown/other* category.

*Comorbidity and medication variables*

Several comorbidity and medication variables were also computed. A modified Elixhauser score was computed using ICD-10 codes in the EHR during the study period with the weighting system defined in van Walraven *et al*. and the R *comorbidity* package, excluding comorbidities with ICD-10 codes not included in our EHR dataset (i.e., AIDS/HIV and mental health conditions) [2,3]. Two additional comorbidity categories were excluded from the modified Elixhauser score: chronic pulmonary disease was excluded as asthma falls under it, and obesity was excluded as we adjusted for BMI separately. We created variables for chronic obstructive pulmonary disease (COPD; J41*, J42*, J43*, J44*) and allergic rhinitis (J30*), choosing to adjust for these comorbidities separately due to their known relationships with asthma. In addition to a primary ICD-10 diagnosis of COPD, we additionally required a prescription for SABA, a short-acting muscarinic antagonist (SAMA), or a combination SABA/SAMA therapy to designate a patient as having COPD (S1 Table). All medication orders during the study period for each patient were used to create a yes/no indicator variable for prescription of inhaled corticosteroid (ICS), a commonly used drug that controls asthma symptoms and prevents exacerbations (S1 Table).

*Asthma exacerbations outcome variable*

Our criteria for asthma exacerbations were based on the U.S. national asthma management guidelines, which recommended oral corticosteroids (OCS) as part of the clinical course for mild, moderate, and severe exacerbations [1]. Exacerbations were defined as encounters with an OCS prescription and either 1) a primary asthma ICD-10 code for encounters with primary diagnosis codes listed or 2) a nonprimary asthma ICD-10 code for encounters without a primary diagnosis listed but only one or two ICD-10 codes listed, which accounted for 53.1% and 17.8%, respectively, of encounters with no primary code.

**SEDH Variables**

*Air pollution exposures*

*NO_2_ and PM2.5.* The datasets we used to obtain measures of these two pollutant variables, which are described in Cooper et al. and van Donkelaar et al., are geophysical-hybrid models, which combine satellite-based measurements, chemical transport modeling, and *in situ* measurements to compute high-resolution pollution estimates [4,5]. The datasets were previously shown to perform well when compared to *in situ* measurements (NO_2_: r = 0.71 for a global model; PM_2.5_: r = 0.82 for North America only) [4,5]. We also validated the NO_2_ dataset previously using another cohort in the Penn Medicine EHR by comparing it to a grid of *in situ* estimates created using inverse distance weighting of EPA regulatory monitor data [6].

*Toxic releases.* Toxics Release Inventory (TRI) datasets for 2017-2020 were downloaded from the EPA TRI website. Cumulative toxic air releases (in kilograms) were summed over the study period for each site located within the spatial extent of Philadelphia with an additional 1-km buffer (spatial region depicted in S3 Fig). After assigning to each patient, TRI was categorized as a yes/no indicator of exposure to toxic releases to account for data sparsity at higher exposure levels.

*Vehicular traffic.* Traffic volume data was downloaded from Pennsylvania Department of Transportation Open Data. Average Daily Vehicle Distance Traveled (DVDT) was computed by multiplying annual average daily traffic (AADT), a measure of the total number of vehicles that travel on a road segment on a typical day, by the length of that segment of road (in kilometers). After assigning to each patient, DVDT was categorized by quartiles as: *Lowest, Low, High,* or *Highest* exposure to account for data sparsity at higher exposure levels.

*Neighborhood socioeconomic environment*

*Area deprivation index (ADI).* The ADI dataset downloaded from Neighborhood Atlas had several Census block groups with missing values due to either low population or high group quarters population. For patients that resided in these block groups, ADI was imputed using random forests (N = 73). After assigning to each patient, we divided the original ADI (a percentile ranging from 1 to 100) by 10 to match the order of magnitude of other numeric variables.

*Built and natural environment*

*Housing code violations*. Housing code violation data for 2017-2020 was downloaded from OpenDataPhilly. Based on the description of each violation code provided by the Philadelphia Department of Licenses and Inspections, violations were classified as “pest-related,” “water damage-related,” and “indoor air contamination-related” based on known environmental asthma triggers. Mold is a well-documented asthma trigger yet it is not regulated by the City of Philadelphia, so water damage was chosen as a proxy [7]. The number of asthma-related violations in each block group was summed and divided by the American Community Survey (ACS) population in that block group to create a normalized neighborhood-level violation count.

*Normalized difference vegetation index*. Atmospherically corrected surface reflectance images for 2017-2020 were downloaded from the Landsat 8 Satellite (Level 2 Collection 2), which captured images at a 30m resolution every 16 days. All images collected during the study period were temporally averaged, excluding images with >20% cloud cover. NDVI was computed in Google Earth Engine [8] using the near infrared (NIR) and red (R) bands and the following formula:

|  | $NDVI=\frac{NIR-R}{NIR+R}$ | (1) |
| --- | --- | --- |

**Modeling Spatial Risk Factors**

The log odds of exacerbations, represented as a binary case-control outcome (0 vs >0 exacerbations), were estimated as a function of location, modeled using a bivariate smooth of latitude and longitude, while simultaneously adjusting for variables over a grid of ~3,000 points across the study region. The spatial smoothing term was evaluated at each pair of latitude and longitude coordinates to produce a map of odds of exacerbations for each point on the grid, then converting from log odds to an odds ratio (OR) using the median odds of exacerbation across all points as a reference. A global test of the null hypothesis that exacerbation odds were not spatially correlated was performed by permuting assignments of cases and controls over patient geocodes 1000 times. A local test for significance was then performed, using the distribution of ORs across all permutations to identify “hotspots” where points ranked in the upper 0.5% and “coldspots” where points ranked in the lower 0.5%.

**Visualizing Geospatial Data**

Visualization of geospatial data and analysis results was conducted in R 4.2 [9] using the R *ggmap* package [10]. Base maps were created using the Stamen Design Toner Lite map tiles from Stadia Maps (<https://docs.stadiamaps.com/map-styles/stamen-toner/>). Use of these Stadia Maps for non-commercial academic use is permitted with appropriate attribution that has been followed. Geographic boundaries were based on the U.S. Census Bureau’s 2019 TIGER/Line shapefiles (<https://catalog.data.gov/dataset/tiger-line-shapefile-2019-2010-nation-u-s-2010-census-urban-area-national>). The TIGER/Line and Census TIGER products are free to use in publications with acknowledgement that the U.S. Census Bureau is their source.

**References**

1. National Asthma Education and Prevention Program. Expert Panel Report 3 (EPR-3): guidelines for the diagnosis and management of asthma-summary report 2007. Bethesda, MD: National Heart, Lung, and Blood Institute (US); 2007.

2. van Walraven C, Austin PC, Jennings A, Quan H, Forster AJ. A modification of the Elixhauser comorbidity measures into a point system for hospital death using administrative data. Medical Care. 2009;47: 626. doi:10.1097/MLR.0b013e31819432e5

3. Gasparini A. Comorbidity: an R package for computing comorbidity scores. J Open Source Softw. 2018;3: 648. doi:10.21105/joss.00648

4. Cooper MJ, Martin RV, McLinden CA, Brook JR. Inferring ground-level nitrogen dioxide concentrations at fine spatial resolution applied to the TROPOMI satellite instrument. Environ Res Lett. 2020;15: 104013. doi:10.1088/1748-9326/aba3a5

5. van Donkelaar A, Hammer MS, Bindle L, Brauer M, Brook JR, Garay MJ, et al. Monthly global estimates of fine particulate matter and their uncertainty. Environ Sci Technol. 2021;55: 15287–15300. doi:10.1021/acs.est.1c05309

6. Schreibman A, Xie S, Hubbard RA, Himes BE. Linking ambient NO2 pollution measures with electronic health record data to study asthma exacerbations. AMIA Jt Summits Transl Sci Proc. 2023;2023: 467–476.

7. Black PN, Udy AA, Brodie SM. Sensitivity to fungal allergens is a risk factor for life-threatening asthma. Allergy. 2000;55: 501–504. doi:10.1034/j.1398-9995.2000.00293.x

8. Ermida SL, Soares P, Mantas V, Göttsche F-M, Trigo IF. Google Earth Engine open-source code for land surface temperature estimation from the Landsat series. Remote Sensing. 2020;12: 1471. doi:10.3390/rs12091471

9. R Core Team (2020). R: a language and environment for statistical computing. Vienna, Austria: R Foundation for Statistical Computing; Available: https://www.R-project.org/

10. Kahle D, Wickham H. ggmap: spatial visualization with ggplot2. The R Journal. 2013;5: 144–161.
