# Supplementary figures and images for: Augmenting electronic health record data with social and environmental determinant of health measures to understand regional factors associated with asthma exacerbations
Source: PLOS Digit Health. 2025 Jun 23;4(6):e0000677. doi: 10.1371/journal.pdig.0000677 (PMC12184914; doi:10.1371/journal.pdig.0000677)

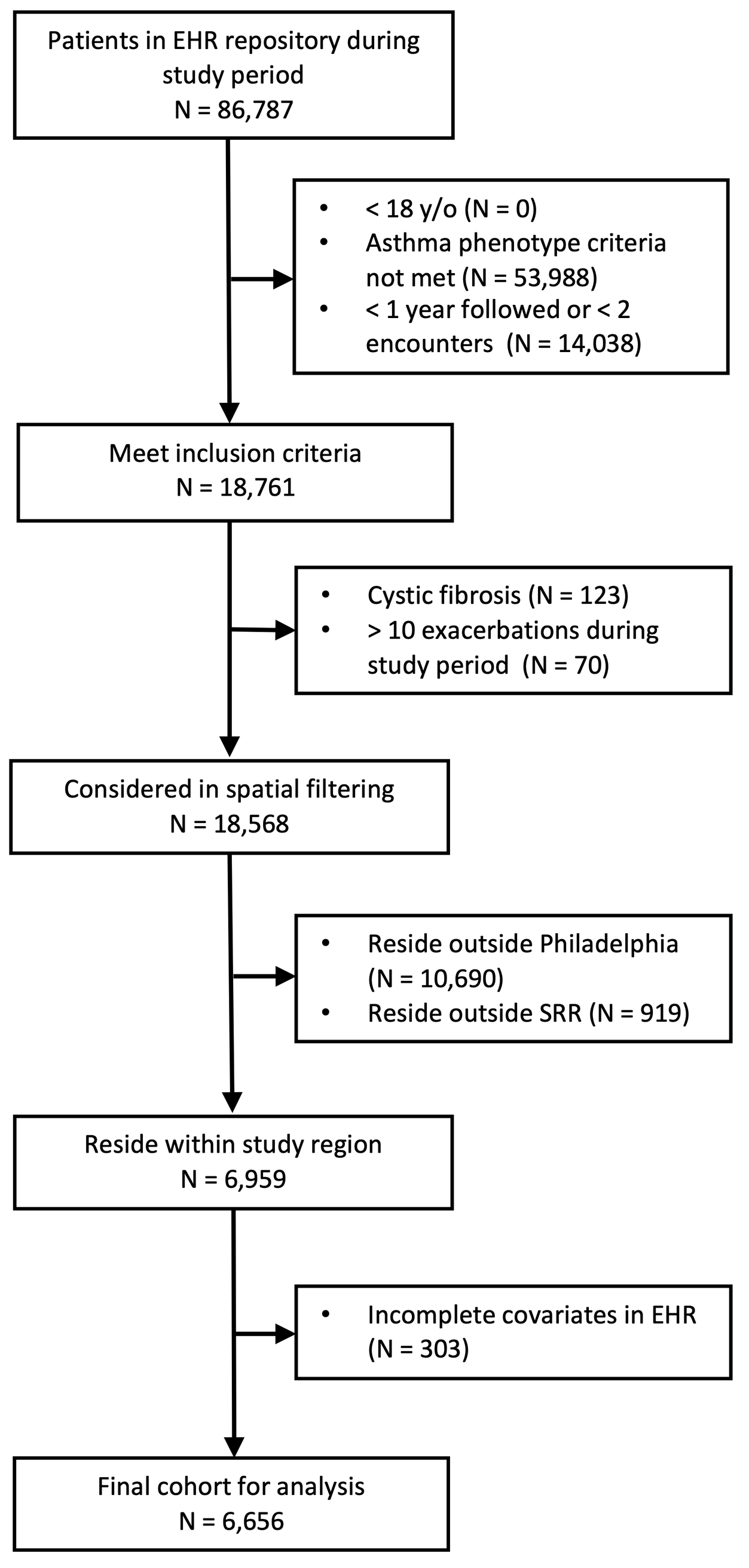

Supplement: S1 Fig — Overview of steps followed to select final patient cohort (N = 6,656) from EHR data on all Penn Medicine patients with at least one asthma ICD code (N = 86,787). (TIF) [file pdig.0000677.s002.tif]

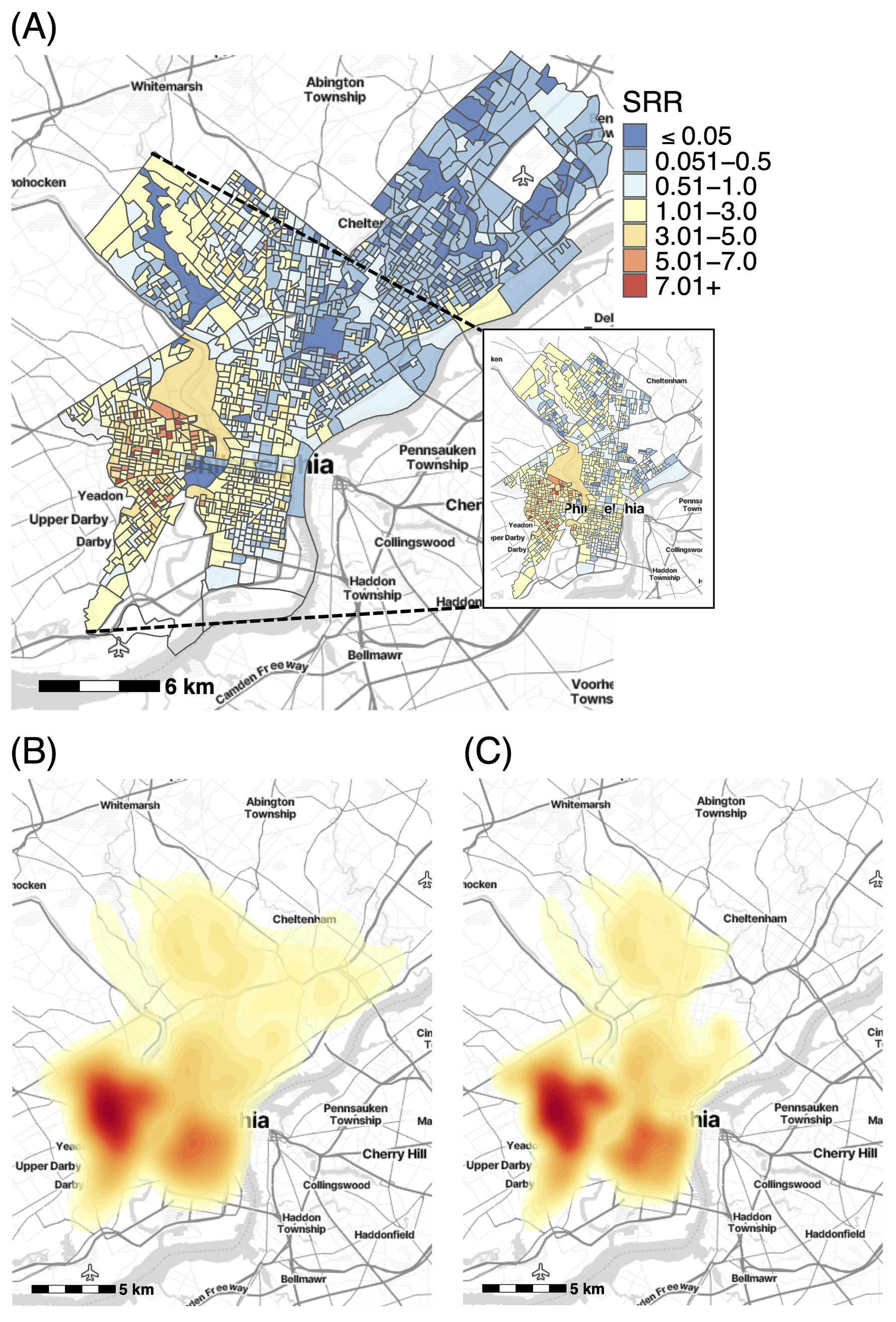

Supplement: S2 Fig — (A) SRR values, defined as the cohort population residing in a block group divided by the underlying population as reported by the 2019 American Community Survey, for all of Philadelphia and for the selected study region (inset box). SRR = 1 indicates no representativeness bias. Density plots of the study cohort (B) before and (C) after filtering for the study region. Base maps were created using the Stamen Design Toner Lite map tiles from Stadia Maps (https://docs.stadiamaps.com/map-styles/stamen-toner/). Geographic boundaries were based on the U.S. Census Bureau’s 2019 TIGER/Line shapefiles (https://catalog.data.gov/dataset/tiger-line-shapefile-2019-2010-nation-u-s-2010-census-urban-area-national). (TIF) [file pdig.0000677.s003.tif]

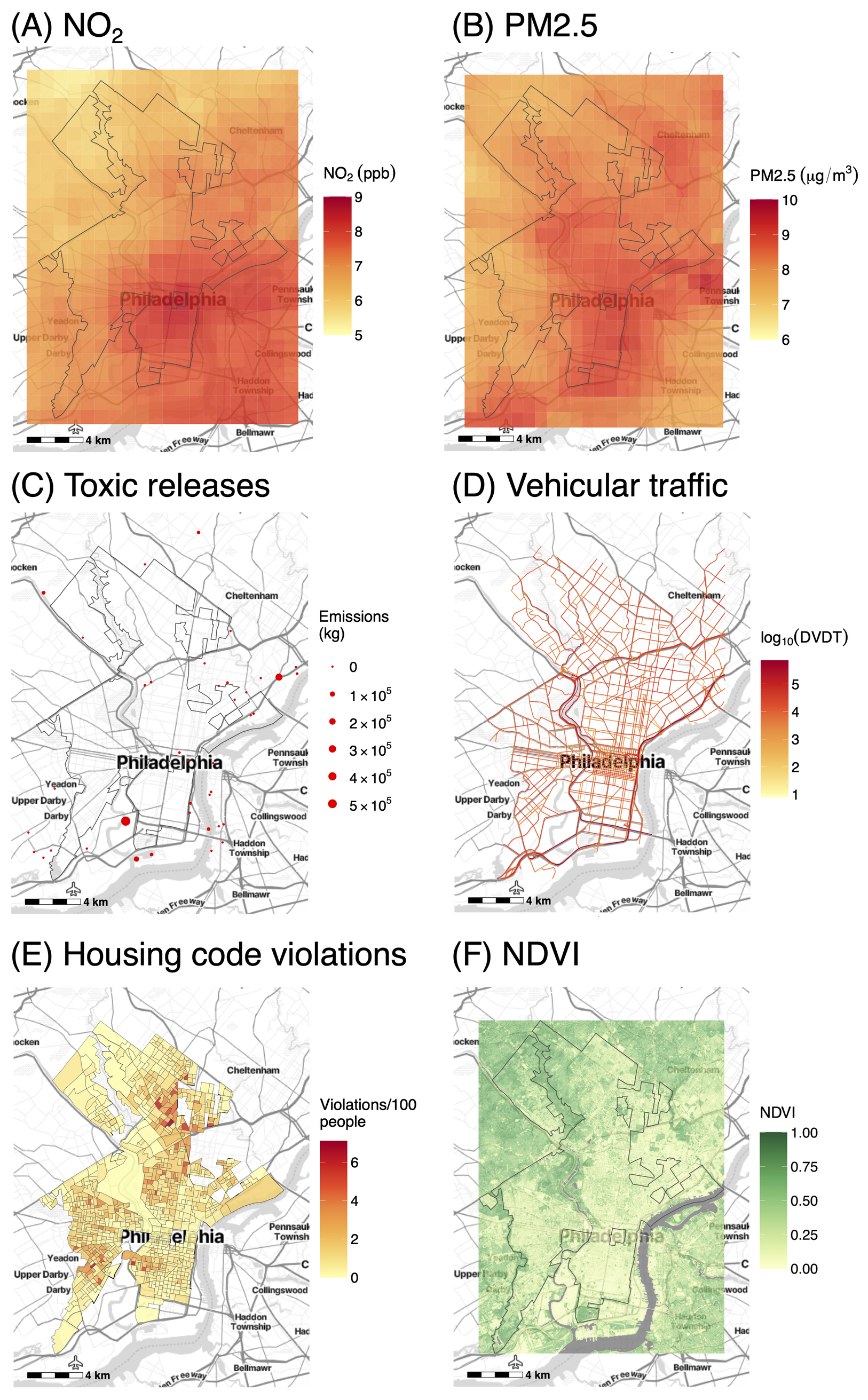

Supplement: S3 Fig — The following maps are shown for the spatial area which comprised our study region: (A) raster of NO2 pollution levels, (B) raster of PM2.5 pollution levels, (C) point sites of toxic releases and the total summed emissions at each site, (D) line segments of roadways and the daily vehicle miles traveled (DVDT) on each, (E) housing violations per block group, normalized by the underlying 2019 American Community Survey population, (F) raster of the normalized difference vegetation index (NDVI). A map of area deprivation index (ADI), which most strongly reduced the spatial variance of odds of exacerbation risk in our spatial GAMs, is shown in Fig 3A. Base maps were created using the Stamen Design Toner Lite map tiles from Stadia Maps (https://docs.stadiamaps.com/map-styles/stamen-toner/). Geographic boundaries were based on the U.S. Census Bureau’s 2019 TIGER/Line shapefiles (https://catalog.data.gov/dataset/tiger-line-shapefile-2019-2010-nation-u-s-2010-census-urban-area-national). (TIF) [file pdig.0000677.s004.tif]

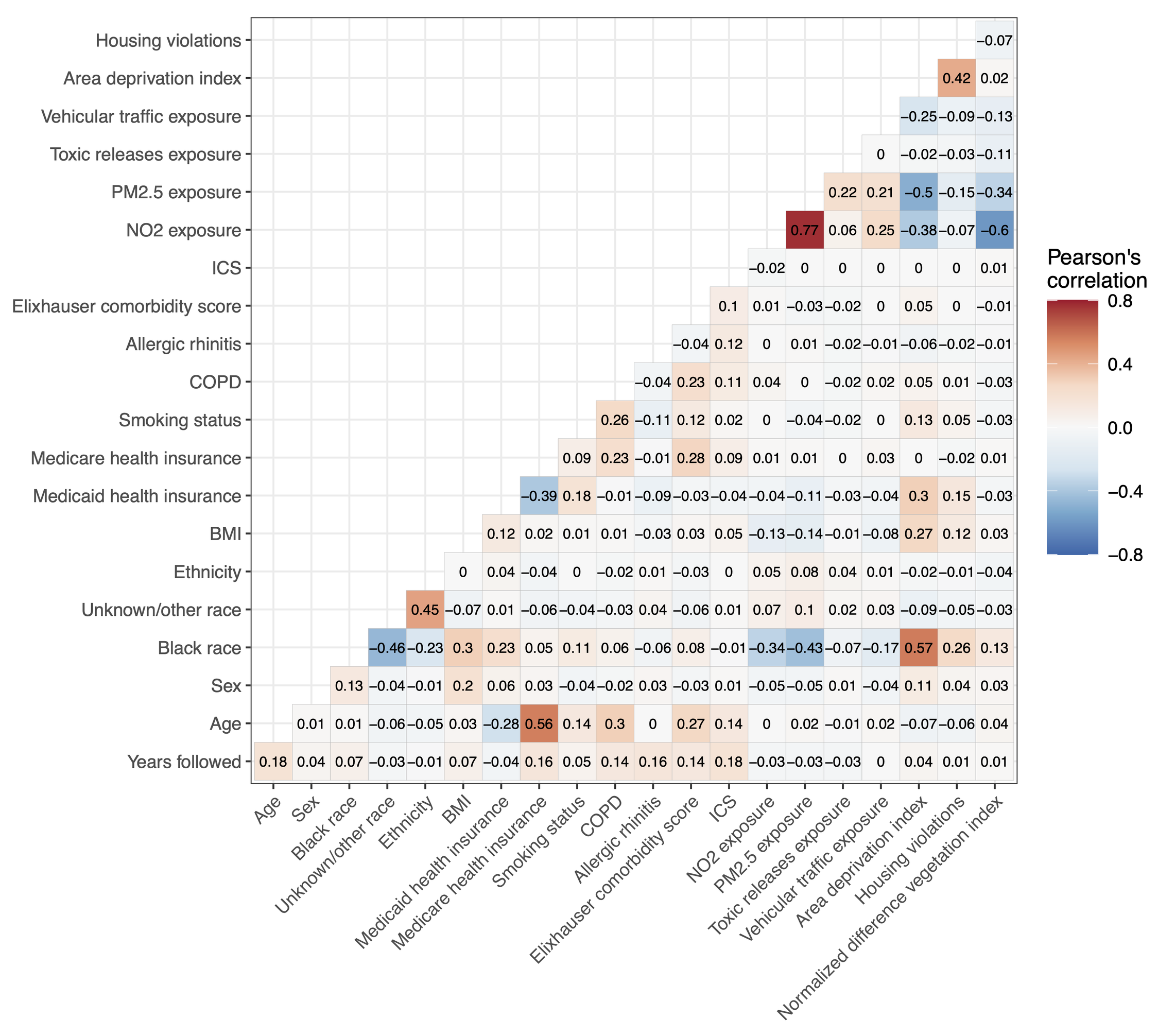

Supplement: S4 Fig — Measures in each box correspond to Pearson’s correlation coefficients. For nominal categorical variables, reference levels are as follows: White (race), Private (health insurance type). (TIF) [file pdig.0000677.s005.tif]

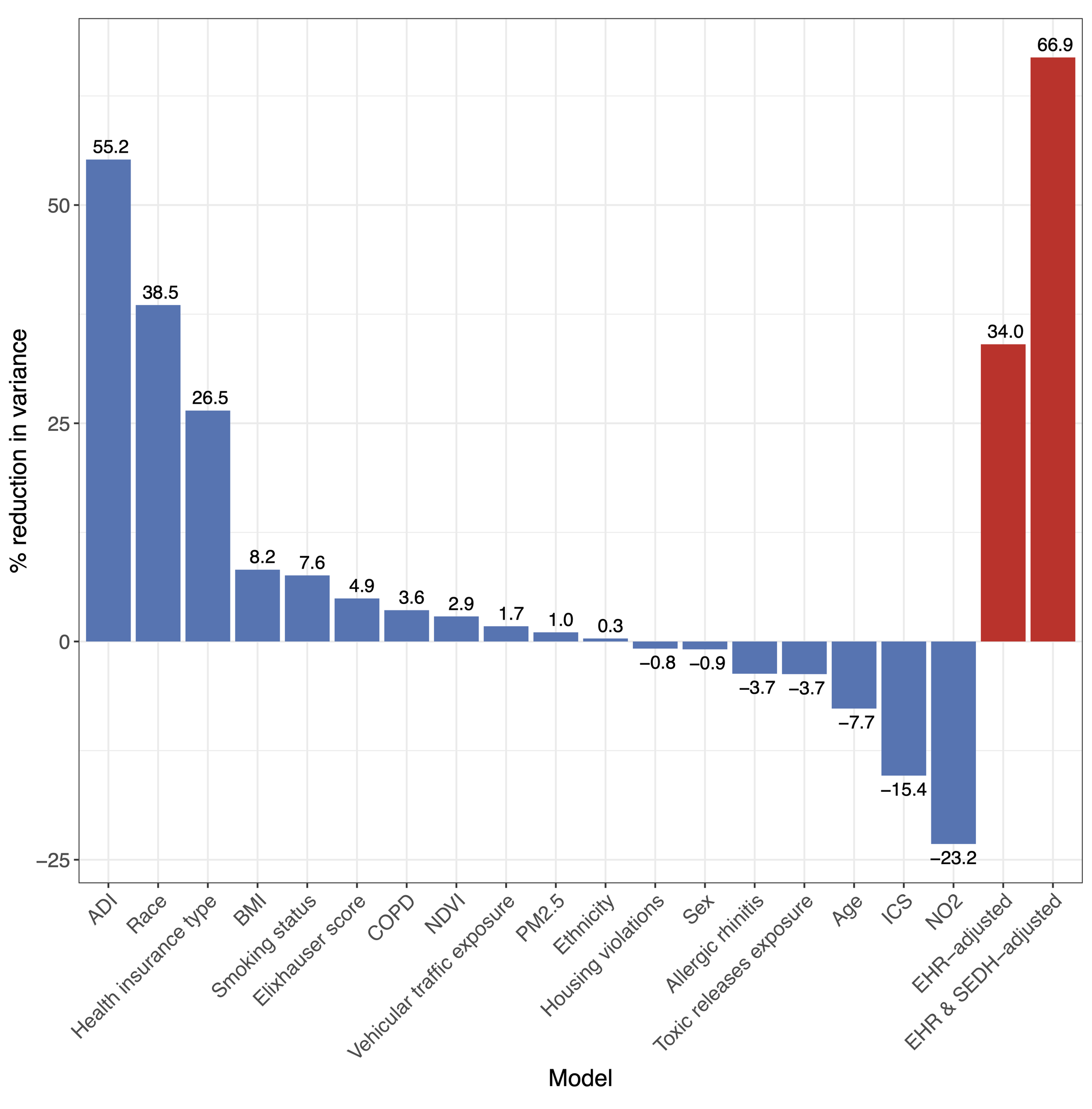

Supplement: S5 Fig — Percent reduction in the variance of ORs across the study region for one-variable-at-a-time adjusted spatial GAMs compared to the unadjusted model. OR changes for models adjusted one variable at a time (in addition to years followed) are shown in blue. Multivariable models (both EHR-adjusted and EHR & SEDH-adjusted) are shown in red for comparison. (TIF) [file pdig.0000677.s006.tif]

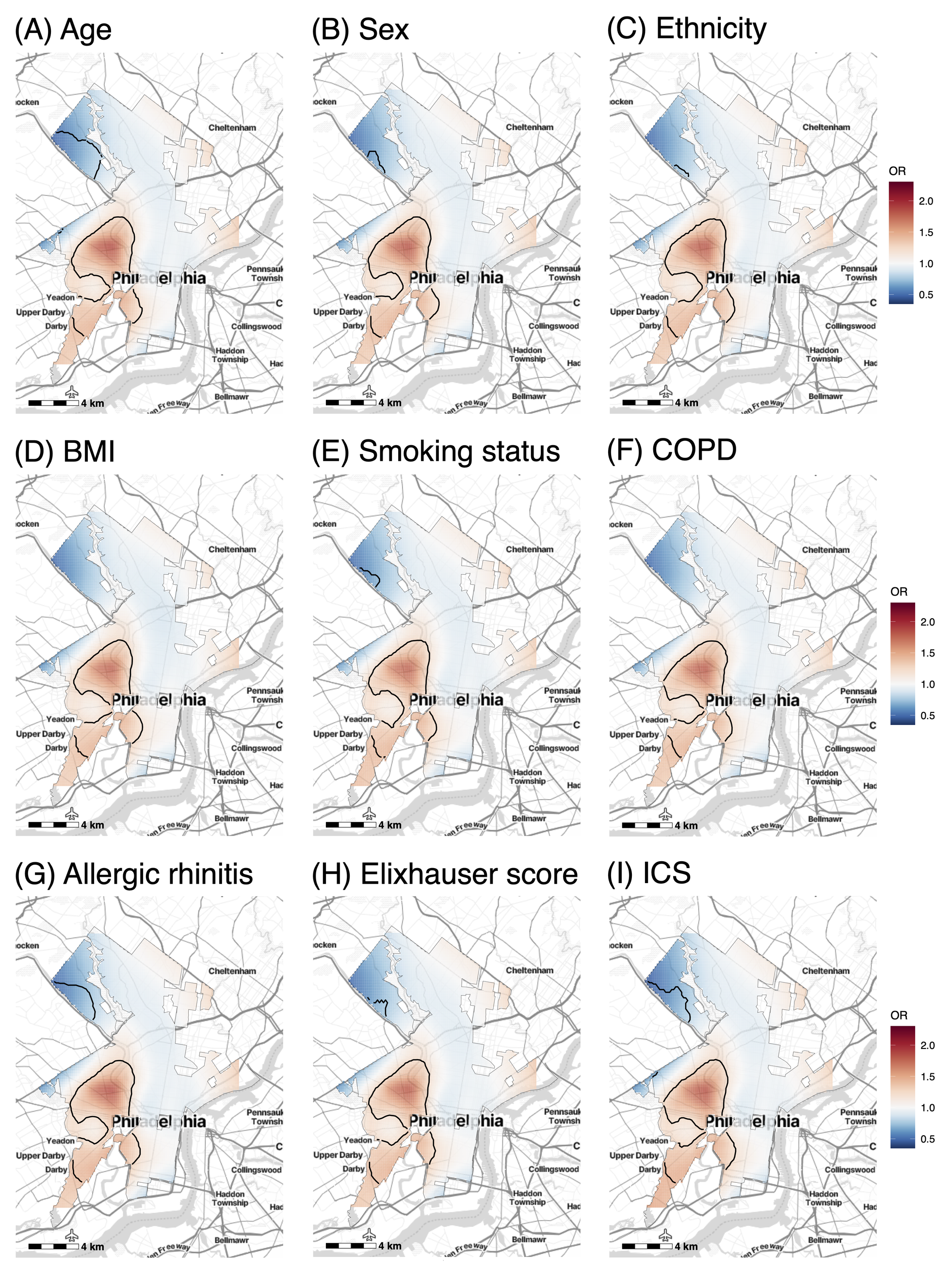

Supplement: S6 Fig — Spatial odds ratios (ORs) of exacerbation are shown after adjusting for years followed and one-at-a-time for the following variables whose percent reduction in variance of ORs was less than 25: (A) age, (B) sex, (C) ethnicity, (D) BMI, (E) smoking status, (F) COPD, (G) allergic rhinitis, (H) Elixhauser comorbidity score, (I) ICS. Base maps were created using the Stamen Design Toner Lite map tiles from Stadia Maps (https://docs.stadiamaps.com/map-styles/stamen-toner/). Geographic boundaries were based on the U.S. Census Bureau’s 2019 TIGER/Line shapefiles (https://catalog.data.gov/dataset/tiger-line-shapefile-2019-2010-nation-u-s-2010-census-urban-area-national). (TIF) [file pdig.0000677.s007.tif]

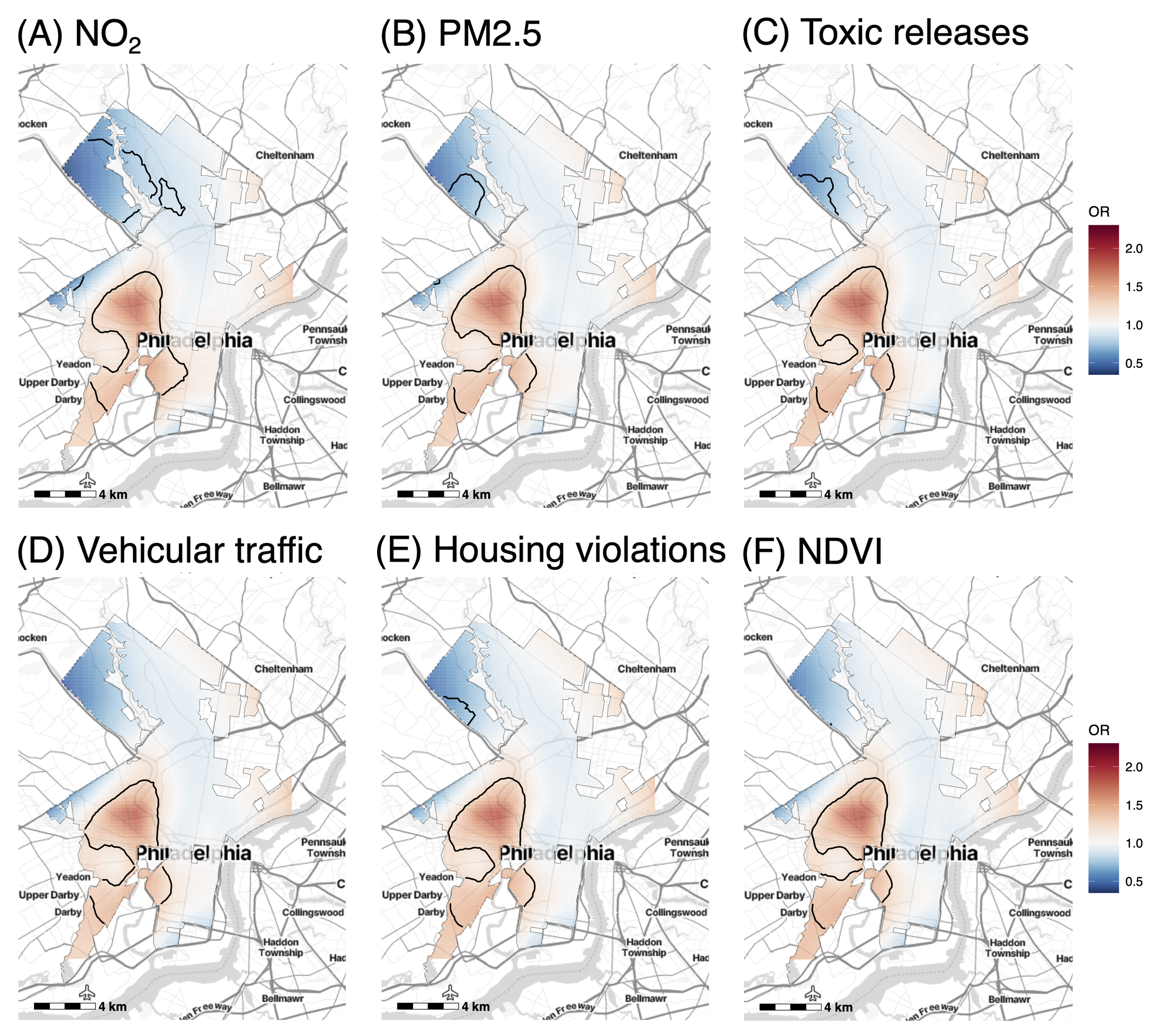

Supplement: S7 Fig — Spatial odds ratios (ORs) of exacerbation are shown after adjusting for years followed and one-at-a-time for the following variables whose reduction in variance of ORs was less than 25: (A) NO2, (B) PM2.5, (C) toxic releases exposure, (D) vehicular traffic, (E) housing violations, (F) normalized difference vegetation index (NDVI). Base maps were created using the Stamen Design Toner Lite map tiles from Stadia Maps (https://docs.stadiamaps.com/map-styles/stamen-toner/). Geographic boundaries were based on the U.S. Census Bureau’s 2019 TIGER/Line shapefiles (https://catalog.data.gov/dataset/tiger-line-shapefile-2019-2010-nation-u-s-2010-census-urban-area-national). (TIF) [file pdig.0000677.s008.tif]

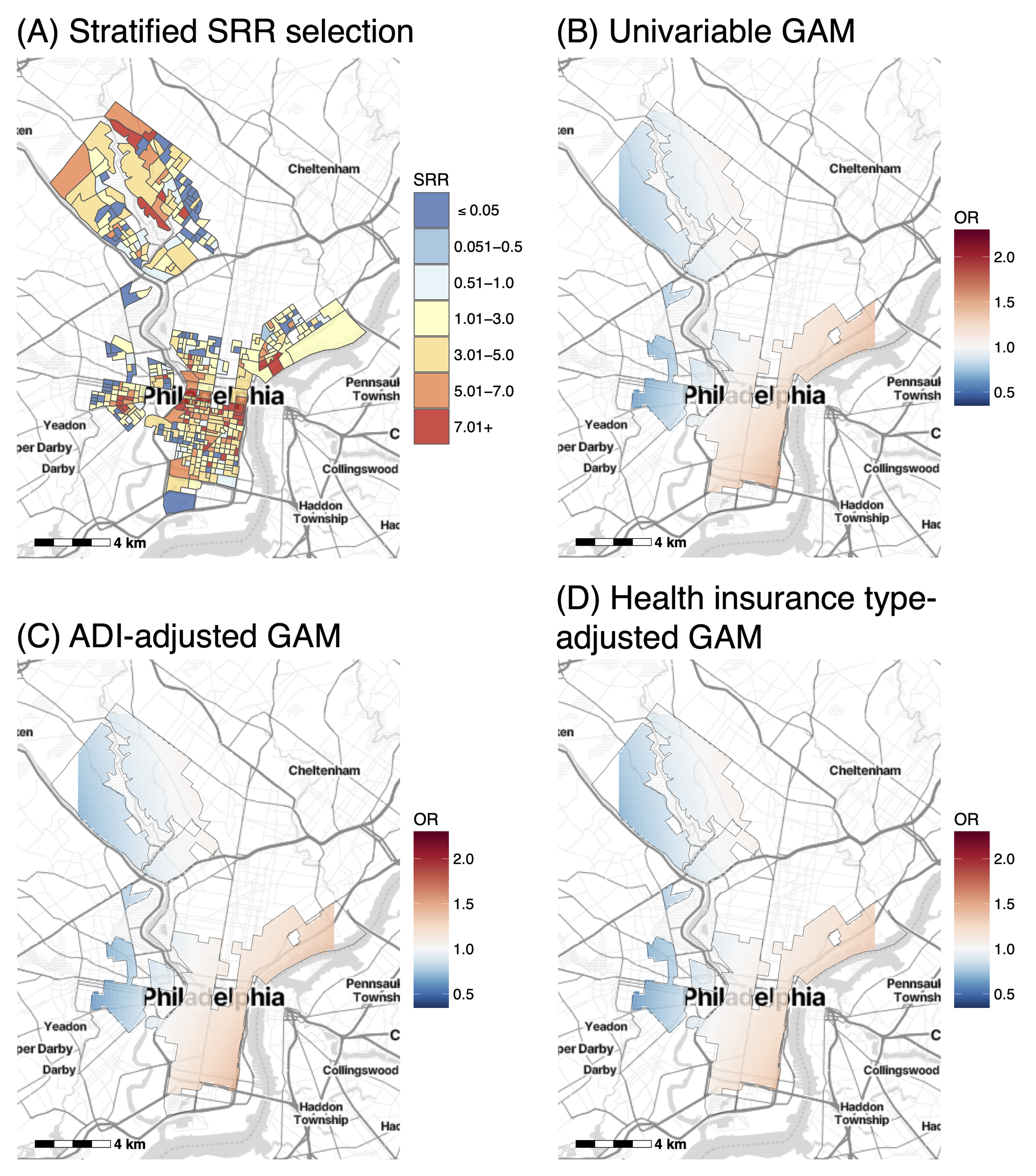

Supplement: S8 Fig — (A) SRR values for the updated study region used in spatial GAMs for patients of White race only (SRR = 1 indicates no representativeness bias). (B) Unadjusted spatial GAM adjusted only for years followed for patients of White race (N = 1,383). Spatial GAMs adjusted additionally for (C) area deprivation index (ADI) and (D) health insurance type. Base maps were created using the Stamen Design Toner Lite map tiles from Stadia Maps (https://docs.stadiamaps.com/map-styles/stamen-toner/). Geographic boundaries were based on the U.S. Census Bureau’s 2019 TIGER/Line shapefiles (https://catalog.data.gov/dataset/tiger-line-shapefile-2019-2010-nation-u-s-2010-census-urban-area-national). (TIF) [file pdig.0000677.s009.tif]
