# Supplementary material for: Augmenting electronic health record data with social and environmental determinant of health measures to understand regional factors associated with asthma exacerbations
Source: PLOS Digit Health. 2025 Jun 23;4(6):e0000677. doi: 10.1371/journal.pdig.0000677 (PMC12184914; doi:10.1371/journal.pdig.0000677)
Supplement: S1 Table — Generic medication names included in medication classes. The following generic drug names recorded in the EHR during the study period were used for asthma and exacerbation phenotyping as well as used as independent variables in select models (i.e., ICS). Instances in which these drugs were listed as investigational or nasal formulations were not included. (DOCX) [file pdig.0000677.s010.docx]

**S1 Table**. **Generic medication names included in medication classes.** The following generic drug names recorded in the EHR during the study period were used for asthma and exacerbation phenotyping as well as used as independent variables in select models (i.e., ICS). Instances in which these drugs were listed as investigational or nasal formulations were not included.

| **Medication class** | **Generic names** |
| --- | --- |
| Short-acting inhaled β_2_-agonist (SABA) | albuterol, albuterol sulfate, levalbuterol HCl, levalbuterol tartrate, metaproterenol sulfate, pirbuterol acetate, albuterol sulfate (sensor) |
| Short-acting muscarinic antagonist (SAMA) | ipratropium bromide, ipratropium bromide HFA |
| SABA/SAMA combination therapy | ipratropium/albuterol |
| Oral or injectable corticosteroid (OCS) | methylprednisolone, methylprednisolone sodium succinate, prednisolone, prednisolone acetate, prednisolone sodium phosphate, prednisone |
| Inhaled corticosteroid (ICS), alone or in combination therapies | beclomethasone diproprionate HFA, beclomethasone diproprionate monohydrate, beclomethasone dipropionate, budesonide, budesonide/glycopyrrolate/formoterol, budesonide/formoterol fumarate, ciclesonide, flunisolide, flunisolide HFA, fluticasone furoate, fluticasone furoate/vilanterol, fluticasone propionate, fluticasone propionate (inhalation route), fluticasone propionate HFA, fluticasone/salmeterol, fluticasone/salmeterol (sensor), fluticasone/umeclidinium/vilanterol, mometasone furoate, mometasone furoate/formoterol fumarate, triamcinolone, triamcinolone acetonide, fluticasone propionate (sensor) |
