# Supplementary material for: Augmenting electronic health record data with social and environmental determinant of health measures to understand regional factors associated with asthma exacerbations
Source: PLOS Digit Health. 2025 Jun 23;4(6):e0000677. doi: 10.1371/journal.pdig.0000677 (PMC12184914; doi:10.1371/journal.pdig.0000677)
Supplement: S2 Table — Sources and spatiotemporal dimensions of geospatial datasets merged with EHR data. (DOCX) [file pdig.0000677.s011.docx]

**S2 Table**. **Sources and spatiotemporal dimensions of geospatial datasets merged with EHR data**.

| **Variable** | **Data source** | **Temporal dimension** | **Original spatial dimension (units)** |
| --- | --- | --- | --- |
| **Air pollution exposures** |  |  |  |
| NO_2_ | Cooper et al., 2022 | 2017-2019 | ~1x1 km (ppbv) |
| PM2.5 | Van Donkelaar et al., 2021 | 2017-2020 | ~1x1 km (μg/m^3^) |
| Toxic releases | EPA TRI*^a^* | 2017-2020 | Point coordinates (pounds of emissions) |
| Vehicular traffic | Pennsylvania DOT*^b^* Open Data Traffic Volumes | 2023 | Line segments (average annual daily traffic) |
| **Neighborhood socioeconomic environment** |  |  |  |
| Area deprivation index | Neighborhood Atlas | 2018 | Census block group (1-100 index) |
| **Built and natural environment** |  |  |  |
| Housing code violations | OpenDataPhilly L&I*^c^* Code Violations | 2017-2020 | Point coordinates (N/A) |
| Normalized difference vegetation index | Google Earth Engine Landsat 8 | 2017-2020 | 30m (-1 – 1 index) |

*^a^*EPA TRI = Environmental Protection Agency Toxics Release Inventory

*^b^*Pennsylvania DOT = Pennsylvania Department of Transportation

*^c^*L&I = Philadelphia Department of Licenses and Inspections
