# Supplementary material for: Augmenting electronic health record data with social and environmental determinant of health measures to understand regional factors associated with asthma exacerbations
Source: PLOS Digit Health. 2025 Jun 23;4(6):e0000677. doi: 10.1371/journal.pdig.0000677 (PMC12184914; doi:10.1371/journal.pdig.0000677)
Supplement: S3 Table — Asthma-related housing code violations extracted from the Philadelphia Department of Licenses and Inspections. (DOCX) [file pdig.0000677.s012.docx]

**S3 Table**. **Asthma-related housing code violations extracted from the Philadelphia Department of Licenses and Inspections.**

| **Code** | **Violation Description** |
| --- | --- |
| 03-306/1 | AIR POLLUTION LO |
| 03-306/2 | AIR POLLUTION LR |
| 14-704/1 | FLOOD PROTECTION |
| 14-704/2 | FLOOD HAZARD |
| A-302.1/1 | FLOOD-SUBSTANTIAL IMPROVEMENT |
| CP-303 | DOWNSPOUTS |
| CP-304 | DRAINS - YARD |
| CP-306 | INFEST RODENTS - EXTERIOR |
| CP-307 | INFEST - SINGLE RES OR NONRES |
| CP-308 | INFEST - MULTI RES OR NONRES |
| CP-327 | INFEST INTERIOR PROPERTY |
| CP-336 | WALL - FOUNDATION |
| CP-337 | COLLARS RODENT ENTRY |
| CP-342 | ROOF - FLASHING AND DRAINAGE |
| CP-349 | BASEMENT ENTRYWAY MAINTAIN |
| PM-302.4/1 | EXT A-GRADING |
| PM-302.4/5 | DRAINAGE-PUMP BASEMENT WATER |
| PM-303.4/1 | INT A-RODENT BURROWS, ELIMINAT |
| PM-303.4/2 | INT A-STORAGE RAISE ITEMS |
| PM-303.5/1 | INT A-EXTERMINATE/MAINTAIN |
| PM-303.5/2 | INT A-EXTERMINATE, TENANT |
| PM-303.5/3 | INT A-EXTERMINATE, OWNER |
| PM-303.5/4 | INT A-EXTERM/MAINTAIN, TENANT |
| PM-304.2/1 | EXT S-FOUNDATION WALLS, MAINTA |
| PM-304.2/2 | EXT S-INSTALL COLLARS |
| PM-304.4/1 | EXT S-ROOF REPAIR |
| PM-304.8/19 | EXT S-WINDOW/FRAME RODENTPROOF |
| PM-304.8/20 | EXT S-DOORS/FRAMES RODENTPROOF |
| PM-304.8/6 | EXT S-BSMNT DOOR/WINDOW/HATCHW |
| PM-305.3/1 | INT S-MAINT SANI/RODENTPROOF |
| PM15-302.2 | EXTERIOR AREA DRAINAGE |
| PM15-302.5 | EXTERIOR AREA RODENT HARBORAGE |
| PM15-304.16 | EXTERIOR STRUCTURE HATCHWAY |
| PM15-304.17 | EXTERIOR STRUCTURE GUARDS |
| PM15-304.1D | EXTERIOR STRUCT UNSAFE COND 4 |
| PM15-304.2 | EXTERIOR STRUCT PROTECTIVE TRE |
| PM15-304.5 | EXTERIOR STRUCTURE FOUNDATIONS |
| PM15-304.6 | EXTERIOR STRUCTURE WALLS |
| PM15-304.7 | EXTERIOR STRUCT ROOF DRAINAGE |
| PM15-309.1 | PEST ELIMINATION INFESTATION |
| PM15-309.3 | PEST ELIM INFEST SGLE OCCUP |
| PM15-309.4 | PEST ELIMINATION INFESTATION |
