# Supplementary material for: Augmenting electronic health record data with social and environmental determinant of health measures to understand regional factors associated with asthma exacerbations
Source: PLOS Digit Health. 2025 Jun 23;4(6):e0000677. doi: 10.1371/journal.pdig.0000677 (PMC12184914; doi:10.1371/journal.pdig.0000677)
Supplement: S4 Table — Patient medications by exacerbation count levels. Shown are the number and percentage of patients receiving each of the medication types listed according to their number of exacerbations during the study period. (DOCX) [file pdig.0000677.s013.docx]

**S4 Table**. **Patient medications by exacerbation count levels.** Shown are the number and percentage of patients receiving each of the medication types listed according to their number of exacerbations during the study period.

| **Medication** | **Number of Exacerbations** | | | | **p-value***^a^* |
| --- | --- | --- | --- | --- | --- |
|  | **0**  N = 4,327 | **1-2**  N = 1,810 | **3-4**  N = 328 | **5+**  N = 191 |  |
| Biologic | 45 (1.0%) | 60 (3.3%) | 25 (7.6%) | 50 (26%) | <10^-4^ |
| ICS*^b^* | 1,820 (42%) | 874 (48%) | 180 (55%) | 124 (65%) | <10^-4^ |
| ICS/LABA | 2,276 (53%) | 1,225 (68%) | 286 (87%) | 179 (94%) | <10^-4^ |
| ICS/LABA/LAMA | 78 (1.8%) | 86 (4.8%) | 20 (6.1%) | 14 (7.3%) | <10^-4^ |
| LABA | 68 (1.6%) | 45 (2.5%) | 16 (4.9%) | 17 (8.9%) | <10^-4^ |
| LABA/LAMA | 76 (1.8%) | 49 (2.7%) | 10 (3.0%) | 6 (3.1%) | 0.032 |
| LAMA | 415 (9.6%) | 282 (16%) | 99 (30%) | 92 (48%) | <10^-4^ |
| Leukotriene modifier | 1,442 (33%) | 838 (46%) | 205 (63%) | 151 (79%) | <10^-4^ |
| OCS | 2,229 (52%) | 1,810 (100%) | 328 (100%) | 191 (100%) | <10^-4^ |
| SABA*^b^* | 4,327 (100%) | 1,810 (100%) | 328 (100%) | 191 (100%) |  |
| SABA/SAMA | 274 (6.3%) | 266 (15%) | 89 (27%) | 69 (36%) | <10^-4^ |
| SAMA | 859 (20%) | 1,022 (56%) | 238 (73%) | 154 (81%) | <10^-4^ |
| Terbutaline | 256 (5.9%) | 99 (5.5%) | 19 (5.8%) | 18 (9.4%) | 0.18 |
| Xanthine | 68 (1.6%) | 60 (3.3%) | 9 (2.7%) | 11 (5.8%) | <10^-4^ |

*^a^*Pearson's Chi-squared test; Fisher's exact test

*^b^*Monotherapy only; combination therapies are shown in separate rows.
