# Supplementary material for: Augmenting electronic health record data with social and environmental determinant of health measures to understand regional factors associated with asthma exacerbations
Source: PLOS Digit Health. 2025 Jun 23;4(6):e0000677. doi: 10.1371/journal.pdig.0000677 (PMC12184914; doi:10.1371/journal.pdig.0000677)
Supplement: S5 Table — Shown are the number and percentage of patients in each level for categorical variables and the Median and Interquartile Range (IQR) for the Years followed variable in complete cases versus those excluded due to missingness in the sex, ethnicity, health insurance type, BMI, and smoking status variables. (DOCX) [file pdig.0000677.s014.docx]

**S5 Table. Characteristics of complete cases and patients excluded due to missingness.** Shown are the number and percentage of patients in each level for categorical variables and the Median and Interquartile Range (IQR) for the Years followed variable in complete cases versus those excluded due to missingness in the sex, ethnicity, health insurance type, BMI, and smoking status variables.

|  | **Complete Case** | |  |
| --- | --- | --- | --- |
| **Characteristic***^a^* | **No**  N = 303 | **Yes**  N = 6,656 | **p-value***^b^* |
| **Exacerbation count** |  |  | 0.76 |
| 0 | 189 (62%) | 4,327 (65%) |  |
| 1-2 | 87 (29%) | 1,810 (27%) |  |
| 3-4 | 18 (5.9%) | 328 (4.9%) |  |
| 5+ | 9 (3.0%) | 191 (2.9%) |  |
| **Years followed** | 2.11 (1.47, 2.97) | 2.77 (1.95, 3.41) | <10^-4^ |
| **Age** |  |  | 1.2x10^-4^ |
| 18-34 | 124 (41%) | 2,155 (32%) |  |
| 35-54 | 118 (39%) | 2,358 (35%) |  |
| 55-74 | 53 (17%) | 1,851 (28%) |  |
| 75+ | 8 (2.6%) | 292 (4.4%) |  |
| **Sex** |  |  | <10^-4^ |
| Male | 118 (39%) | 1,539 (23%) |  |
| Female | 185 (61%) | 5,117 (77%) |  |
| **Race** |  |  | 0.0017 |
| White | 64 (21%) | 1,456 (22%) |  |
| Black | 195 (64%) | 4,628 (70%) |  |
| Unknown/Other | 44 (15%) | 572 (8.6%) |  |
| **Ethnicity** |  |  | 0.054 |
| Non-Hispanic/Latino | 236 (99%) | 6,417 (96%) |  |
| Hispanic/Latino | 3 (1.3%) | 239 (3.6%) |  |
| Unknown | 64 | 0 |  |
| **BMI** |  |  | 0.062 |
| Not Overweight or Obese | 44 (27%) | 1,291 (19%) |  |
| Overweight | 43 (26%) | 1,554 (23%) |  |
| Class 1 Obesity | 34 (21%) | 1,460 (22%) |  |
| Class 2 Obesity | 19 (12%) | 1,028 (15%) |  |
| Class 3 Obesity | 24 (15%) | 1,323 (20%) |  |
| Unknown | 139 | 0 |  |
| **Health insurance type** |  |  | 1.5x10^-4^ |
| Private | 89 (40%) | 2,891 (43%) |  |
| Medicaid | 96 (44%) | 2,095 (31%) |  |
| Medicare | 35 (16%) | 1,670 (25%) |  |
| Unknown | 83 | 0 |  |
| **Smoking status** |  |  | 0.081 |
| Never Smoked | 127 (61%) | 3,781 (57%) |  |
| Ever Smoker | 63 (30%) | 1,937 (29%) |  |
| Current Smoker | 18 (8.7%) | 938 (14%) |  |
| Unknown | 95 | 0 |  |
| **COPD** | 13 (4.3%) | 691 (10%) | 5.8x10^-4^ |
| **Allergic rhinitis** | 57 (19%) | 2,351 (35%) | <10^-4^ |
| **Elixhauser comorbidity score** |  |  | <10^-4^ |
| <0 | 3 (1.0%) | 331 (5.0%) |  |
| 0 | 261 (86%) | 4,541 (68%) |  |
| 1-9 | 30 (9.9%) | 1,210 (18%) |  |
| 10+ | 9 (3.0%) | 574 (8.6%) |  |
| **ICS** | 159 (52%) | 5,060 (76%) | <10^-4^ |

*^a^*Units: age (years), ICS (yes/no indicator of inhaled corticosteroid prescription). See Methods for more details.

*^b^*Pearson's Chi-squared test; Wilcoxon rank sum test
