# Supplementary material for: Augmenting electronic health record data with social and environmental determinant of health measures to understand regional factors associated with asthma exacerbations
Source: PLOS Digit Health. 2025 Jun 23;4(6):e0000677. doi: 10.1371/journal.pdig.0000677 (PMC12184914; doi:10.1371/journal.pdig.0000677)
Supplement: S6 Table — (DOCX) [file pdig.0000677.s015.docx]

**S6 Table. Adjusted Generalized Variance Inflation Factors (GVIFs) for each EHR and SEDH variable included in the EHR & SEDH-adjusted negative binomial and logistic regression models.**

| **Predictor** | **Adjusted GVIF***^a^* |
| --- | --- |
| Years followed | 1.06 |
| Age | 1.11 |
| Sex | 1.04 |
| Race | 1.22 |
| Ethnicity | 1.13 |
| BMI | 1.03 |
| Health insurance type | 1.18 |
| Smoking status | 1.06 |
| COPD | 1.11 |
| Allergic rhinitis | 1.04 |
| Elixhauser comorbidity score | 1.04 |
| ICS | 1.03 |
| NO_2_ exposure | 1.97 |
| PM2.5 exposure | 1.79 |
| Toxic releases exposure | 1.06 |
| Vehicular traffic exposure | 1.02 |
| Area deprivation index | 1.49 |
| Housing violations | 1.11 |
| Normalized difference vegetation index | 1.32 |
