# Supplementary material for: Augmenting electronic health record data with social and environmental determinant of health measures to understand regional factors associated with asthma exacerbations
Source: PLOS Digit Health. 2025 Jun 23;4(6):e0000677. doi: 10.1371/journal.pdig.0000677 (PMC12184914; doi:10.1371/journal.pdig.0000677)
Supplement: S7 Table — Individual-level asthma exacerbation risk factors in multivariable negative binomial regression models. Shown are the adjusted incidence rate ratios (IRRs), 95% confidence intervals (CIs), and p-values for negative binomial models of asthma exacerbations as a count outcome adjusted for EHR-derived variables only and for both EHR-derived and SEDH variables. (DOCX) [file pdig.0000677.s016.docx]

**S7 Table**. **Individual-level asthma exacerbation risk factors in multivariable negative binomial regression models.** Shown are the adjusted incidence rate ratios (IRRs), 95% confidence intervals (CIs), and p-values for negative binomial models of asthma exacerbations as a count outcome adjusted for EHR-derived variables only and for both EHR-derived and SEDH variables.

|  | **EHR-adjusted** | | | **EHR & SEDH-adjusted** | | |
| --- | --- | --- | --- | --- | --- | --- |
| **Characteristic***^a^* | **IRR** | **95% CI** | **p-value** | **IRR** | **95% CI** | **p-value** |
| **Age** |  |  |  |  |  |  |
| 18-34 | — | — |  | — | — |  |
| 35-54 | 1.12 | 1.00, 1.26 | 0.050 | 1.13 | 1.00, 1.26 | 0.039 |
| 55-74 | 0.93 | 0.81, 1.08 | 0.35 | 0.94 | 0.81, 1.08 | 0.39 |
| 75+ | 1.02 | 0.79, 1.32 | 0.88 | 1.04 | 0.81, 1.35 | 0.76 |
| **Sex** |  |  |  |  |  |  |
| Male | — | — |  | — | — |  |
| Female | 0.88 | 0.79, 0.98 | 0.028 | 0.88 | 0.79, 0.98 | 0.019 |
| **Race** |  |  |  |  |  |  |
| White | — | — |  | — | — |  |
| Black | 1.47 | 1.30, 1.67 | <10^-4^ | 1.48 | 1.27, 1.72 | <10^-4^ |
| Unknown/Other | 1.11 | 0.90, 1.37 | 0.35 | 1.11 | 0.89, 1.38 | 0.35 |
| **Ethnicity** |  |  |  |  |  |  |
| Non-Hispanic/Latino | — | — |  | — | — |  |
| Hispanic/Latino | 0.79 | 0.58, 1.05 | 0.11 | 0.77 | 0.57, 1.04 | 0.081 |
| **BMI** |  |  |  |  |  |  |
| Not Overweight or Obese | — | — |  | — | — |  |
| Overweight | 1.11 | 0.96, 1.28 | 0.16 | 1.11 | 0.97, 1.28 | 0.14 |
| Class 1 Obesity | 0.96 | 0.83, 1.11 | 0.58 | 0.97 | 0.83, 1.12 | 0.64 |
| Class 2 Obesity | 1.07 | 0.91, 1.26 | 0.39 | 1.07 | 0.91, 1.26 | 0.42 |
| Class 3 Obesity | 1.11 | 0.95, 1.29 | 0.20 | 1.11 | 0.95, 1.29 | 0.18 |
| **Health insurance type** |  |  |  |  |  |  |
| Private | — | — |  | — | — |  |
| Medicaid | 1.34 | 1.20, 1.50 | <10^-4^ | 1.31 | 1.17, 1.47 | <10^-4^ |
| Medicare | 1.02 | 0.89, 1.17 | 0.78 | 1.01 | 0.88, 1.15 | 0.92 |
| **Smoking status** |  |  |  |  |  |  |
| Never Smoked | — | — |  | — | — |  |
| Ever Smoker | 1.09 | 0.98, 1.21 | 0.14 | 1.08 | 0.98, 1.21 | 0.13 |
| Current Smoker | 1.15 | 1.00, 1.32 | 0.058 | 1.14 | 0.99, 1.31 | 0.059 |
| **COPD** |  |  |  |  |  |  |
| No | — | — |  | — | — |  |
| Yes | 1.04 | 0.90, 1.21 | 0.63 | 1.03 | 0.89, 1.20 | 0.72 |
| **Allergic rhinitis** |  |  |  |  |  |  |
| No | — | — |  | — | — |  |
| Yes | 1.11 | 1.01, 1.21 | 0.039 | 1.11 | 1.01, 1.22 | 0.026 |
| **Elixhauser comorbidity score** |  |  |  |  |  |  |
| <0 | — | — |  | — | — |  |
| 0 | 1.26 | 1.02, 1.55 | 0.038 | 1.26 | 1.02, 1.56 | 0.034 |
| 1-9 | 1.51 | 1.20, 1.90 | 4.9x10^-4^ | 1.51 | 1.20, 1.90 | 3.8x10^-4^ |
| 10+ | 1.27 | 0.99, 1.64 | 0.071 | 1.27 | 0.98, 1.63 | 0.069 |
| **ICS** |  |  |  |  |  |  |
| No | — | — |  | — | — |  |
| Yes | 2.31 | 2.04, 2.63 | <10^-4^ | 2.32 | 2.05, 2.64 | <10^-4^ |
| **NO_2_ exposure** |  |  |  | 1.13 | 0.98, 1.31 | 0.094 |
| **PM2.5 exposure** |  |  |  | 1.01 | 0.85, 1.21 | 0.87 |
| **Toxic releases exposure** |  |  |  |  |  |  |
| No |  |  |  | — | — |  |
| Yes |  |  |  | 1.04 | 0.83, 1.29 | 0.75 |
| **Vehicular traffic exposure** |  |  |  |  |  |  |
| Lowest |  |  |  | — | — |  |
| Low |  |  |  | 1.00 | 0.88, 1.14 | 0.99 |
| High |  |  |  | 1.05 | 0.92, 1.19 | 0.47 |
| Highest |  |  |  | 0.93 | 0.81, 1.06 | 0.26 |
| **Area deprivation index** |  |  |  | 1.02 | 1.00, 1.05 | 0.064 |
| **Housing violations** |  |  |  | 0.95 | 0.91, 1.00 | 0.045 |
| **Normalized difference vegetation index** |  |  |  | 0.95 | 0.47, 1.93 | 0.89 |
| **AIC** | 14,544 |  |  | 14,546 |  |  |

*^a^*Units are as follows: age (years), ICS (yes/no indicator of inhaled corticosteroid prescription), NO_2_ (ppbv), PM2.5 (μg/m^3^), toxic releases exposure (yes/no indicator of exposure), area deprivation index (unitless index scaled by dividing by 10), housing violations (housing violations per 100 people), normalized difference vegetation index (unitless index ranging from -1 to 1). See Methods for more details.
