# Supplementary material for: Augmenting electronic health record data with social and environmental determinant of health measures to understand regional factors associated with asthma exacerbations
Source: PLOS Digit Health. 2025 Jun 23;4(6):e0000677. doi: 10.1371/journal.pdig.0000677 (PMC12184914; doi:10.1371/journal.pdig.0000677)
Supplement: S8 Table — Shown are the adjusted odds ratios (ORs), 95% confidence intervals (CIs), and p-values for spatial GAMs of asthma exacerbations as a dichotomous outcome adjusted for years followed and one-at-a-time for ADI, race, and health insurance type, the three variables whose percent reduction in variance of ORs was greater than 25. (DOCX) [file pdig.0000677.s017.docx]

**S8 Table. Spatial GAMs of asthma exacerbations adjusted for individual risk factors that most changed risk**. Shown are the adjusted odds ratios (ORs), 95% confidence intervals (CIs), and p-values for spatial GAMs of asthma exacerbations as a dichotomous outcome adjusted for years followed and one-at-a-time for ADI, race, and health insurance type, the three variables whose percent reduction in variance of ORs was greater than 25.

|  | **ADI-adjusted** | | | **Race-adjusted** | | | **Health insurance type-adjusted** | | |
| --- | --- | --- | --- | --- | --- | --- | --- | --- | --- |
| **Characteristic***^a^* | **OR** | **95% CI** | **p-value** | **OR** | **95% CI** | **p-value** | **OR** | **95% CI** | **p-value** |
| **Years followed** | 1.27 | 1.20, 1.35 | <10^-4^ | 1.26 | 1.19, 1.34 | <10^-4^ | 1.28 | 1.20, 1.36 | <10^-4^ |
| **ADI** | 1.05 | 1.03, 1.07 | <10^-4^ |  |  |  |  |  |  |
| **Race** |  |  |  |  |  |  |  |  |  |
| White |  |  |  | — | — | — |  |  |  |
| Black |  |  |  | 1.66 | 1.44, 1.91 | <10^-4^ |  |  |  |
| Unknown/  Other |  |  |  | 1.09 | 0.88, 1.36 | 0.43 |  |  |  |
| **Health insurance type** |  |  |  |  |  |  |  |  |  |
| Private |  |  |  |  |  |  | — | — | — |
| Medicaid |  |  |  |  |  |  | 1.30 | 1.15, 1.46 | <10^-4^ |
| Medicare |  |  |  |  |  |  | 1.05 | 0.92, 1.20 | 0.46 |
| **AIC** | 8,502 |  |  | 8,471 |  |  | 8,501 |  |  |

*^a^*Units are as follows: area deprivation index (unitless index scaled by dividing by 10). See Methods for more details.
